# Selection and Validation of Reference Genes for mRNA Expression by Quantitative Real-Time PCR Analysis in *Neolamarckia cadamba*

**DOI:** 10.1038/s41598-018-27633-5

**Published:** 2018-06-18

**Authors:** Tian Huang, Jianmei Long, Si-Wen Liu, Zi-Wei Yang, Qi-Jin Zhu, Xiao-Lan Zhao, Changcao Peng

**Affiliations:** 1 State Key Laboratory for Conservation and Utilization of Subtropical Agro-bioresources, South China Agricultural University, Guangzhou, 510642 China; 2 Guangdong Key Laboratory for Innovative Development and Utilization of Forest Plant Germplasm, College of Forestry and Landscape Architecture, South China Agricultural University, Guangzhou, 510642 China

## Abstract

*Neolamarckia cadamba* is an economically-important fast-growing tree species in South China and Southeast Asia. As a prerequisite first step for future gene expression studies, we have identified and characterized a series of stable reference genes that can be used as controls for quantitative real time PCR (qRT-PCR) expression analysis in this study. The expression stability of 15 candidate reference genes in various tissues and mature leaves under different conditions was evaluated using four different algorithms, i.e., geNorm, NormFinder, BestKeeper and RefFinder. Our results showed that *SAMDC* was the most stable of the selected reference genes across the set of all samples, mature leaves at different photosynthetic cycles and under drought stress, whereas *RPL10A* had the most stable expression in various tissues. *PGK* and *RPS25* were considered the most suitable reference for mature leaves at different developmental stages and under cold treatment, respectively. Additionally, the gene expression profiles of sucrose transporter 4 (NcSUT4), and 9‐cis‐epoxycarotenoid dioxygenase 3 (NcNCED3) were used to confirm the validity of candidate reference genes. Collectively, our study is the first report to validate the optimal reference genes for normalization under various conditions in *N*. *cadamba* and will benefit the future discovery of gene function in this species.

## Introduction

Quantitative real-time polymerase chain reaction (qRT-PCR) is a powerful tool which monitors the entire PCR process in real-time. Currently, northern blot, microarray, qRT-PCR and high-throughput sequencing are the four common methods for gene expression analysis. Among them, qRT-PCR is most widely used for accurate quantification of gene expression because of its high sensitivity, accuracy, specificity and reproducibility and low cost^1–3^. However, the accuracy of gene expression is easily affected by the factors including RNA integrity, reverse transcription reaction efficiency, cDNA quality and genomic DNA contamination^4–7^. Hence, normalization is an essential step in qRT-PCR assay^5^. Among the multiple strategies proposed^8^, the use of reference gene is the most popular method for data normalization^9^. Udvardi *et al*.^10^ have illustrated eleven golden rules of qRT-PCR, among which a suitable reference gene should be stably expressed in the samples under a range of given experimental conditions, so as to ensure the accuracy and reproducibility of measurement in gene expression^11^. In contrast, inappropriate reference gene selection can lead to discrepancies in interpreting the qRT-PCR data, potentially leading to inappropriate conclusions regarding expression of target genes. Therefore, selection of appropriate reference genes is of great importance for data normalization in qRT-PCR analysis.

Numerous studies have indicated that the housekeeping genes involved in maintaining the basic metabolism of cell, such as *ACT* (*Actin*), *TUA* (*Alpha-tubulin*), *GAPDH* (*Glyceraldehyde 3-phosphate dehydrogenase*) and *18S rRNA*(*18S ribosomal RNA*) have been routinely used as internal controls in qRT-PCR and as the reference genes for standardized analysis in many plants species^12–14^. They were assumed to be stably expressed in various tissues at different developmental stage and under a wide range of conditions^15^. However, several recent reports showed that these traditional reference genes are not always stable in some specific conditions^16,17^. In addition, there is no reference gene universally expressed at constant level across all species under wide range of experimental conditions^18^. Thus, it is essential to systematically validate the stability of potential reference genes before their use in qRT-PCR assays in any new experimental organism.

Statistical algorithms such as geNorm^1^, NormFinder^19^ and Bestkeepeer^20^ have been specifically developed for the evaluation of candidate reference genes stability and determination of the best reference in one or several specific experimental conditions. By using these programs, reference gene validation has been successfully carried out in many plant species, including *Arabidopsis*^21^, rice^15^, maize^22^, soybean^23^, radish^9^, strawberry^24^, orchardgrass^7^, *Lycoris aurea*^25^, banana^5^, citrus^26^, grape^27^, poplar^28^, litchi^29^ and longan^30^. The reference genes identified in each species have been frequently used in the subsequent studies, which accelerated to explore the mechanisms of key biological processes in plant species.

*Neolamarckia cadamba*, a member of the Rubiaceae family, is widely distributed in South China and South Asia, and is cultivated due to its inherent economic value in multiple aspects^31^. It has been praised as “a miraculous tree” since the World Forest Congress in 1972 due to its fast growth^32,33^, reaching heights of 17 m and trunk diameters of 25 cm within 9 years of growth under normal conditions^34^. Therefore, it is a good choice to use *N*. *cadamba* for the forest rehabilitation in tropical regions. In addition, *N*. *cadamba* lumber is also a useful source for raw materials in paper production, furniture, building and biomass utilization. *N*. *cadamba* has also attracted lots of attention on the medicinal value including application in the treatment of various ailments and extraction of bioactive compounds^35^. Recently, *N*. *cadamba* was used as model plant to study the xylogenesis during wood formation^31^, although these studies were primarily physiological in nature, and did not include molecular details such as transcriptional regulation of these processes. In order to explore the mechanism of wood formation and fast growth in *N*. *cadamba*, gene expression analysis is one of the most important steps. However, so far there is no report on the identification of reference genes for normalization in gene expression detection in *N*. *cadamba*.

To identify suitable reference genes for accurate quantification of target genes in *N*. *cadamba*, fifteen potential reference genes including Actin 1 (ACT1), Actin 7 (ACT7), Actin 11 (ACT11), Tubulin alpha 2 (TUA2), Tubulin alpha 4 (TUA4), Tubulin alpha 5 (TUA5), Elongation factor 1-alpha (EF-1-α), Ribulose bisphosphate carboxylase (Rubisco), Ribosomal protein S25 (RPS25), Ribosomal protein L10A (RPL10A), Malate dehydrogenase (MDH), Glyceraldehyde-3-phosphate dehydrogenase (GAPDH), Phosphoglycerate kinase (PGK), S-adenosylmethionine decarboxylase (SAMDC), F-Box protein (F-BOX) were selected, and their sequences retrieved from our previously generated transcriptome data^31^. The expression stability of the selected candidates was evaluated by qRT-PCR using a set of cDNAs from 22 different samples of *N*. *cadamba*, which included various tissues, mature leaves at different developmental stages and photosynthetic cycles, and under cold and drought treatments. Optimal reference genes were suggested for each experimental condition by using four different gene expression analysis programs. Furthermore, sucrose transporter 4 (*NcSUT4*), responsible for sucrose transportation, and 9-cis-epoxycarotenoid dioxygenase 3 (*NcNCED3*), a key enzyme involved in the synthesis of ABA, were used to confirm the reliability and effectiveness of the selected reference genes. Our study provided a list of suitable reference genes for normalization of qRT-PCR analyses, which is anticipated to facilitate the gene expression analyses in *N*. *cadamba*.

## Results

### Primers specificity, PCR efficiencies and expression profile of the candidate reference genes

A single PCR product from each primer pair of candidate reference genes was amplified with expected size by agarose gel electrophoresis analysis (see Supplementary Fig. S1). Specific amplifications were also confirmed by melting curves with a single peak in each candidate reference genes (see Supplementary Fig. S2). Standard curves were generated using serial dilution series, and high linear correlations (R^2^ > 0.99) were detected for in all genes (see Supplementary Table S1). The PCR amplification efficiencies for fifteen genes varied from 94.5% for *ACT1* to 116.3% for *RPS25* (see Supplementary Table S1).

**Figure 1:**
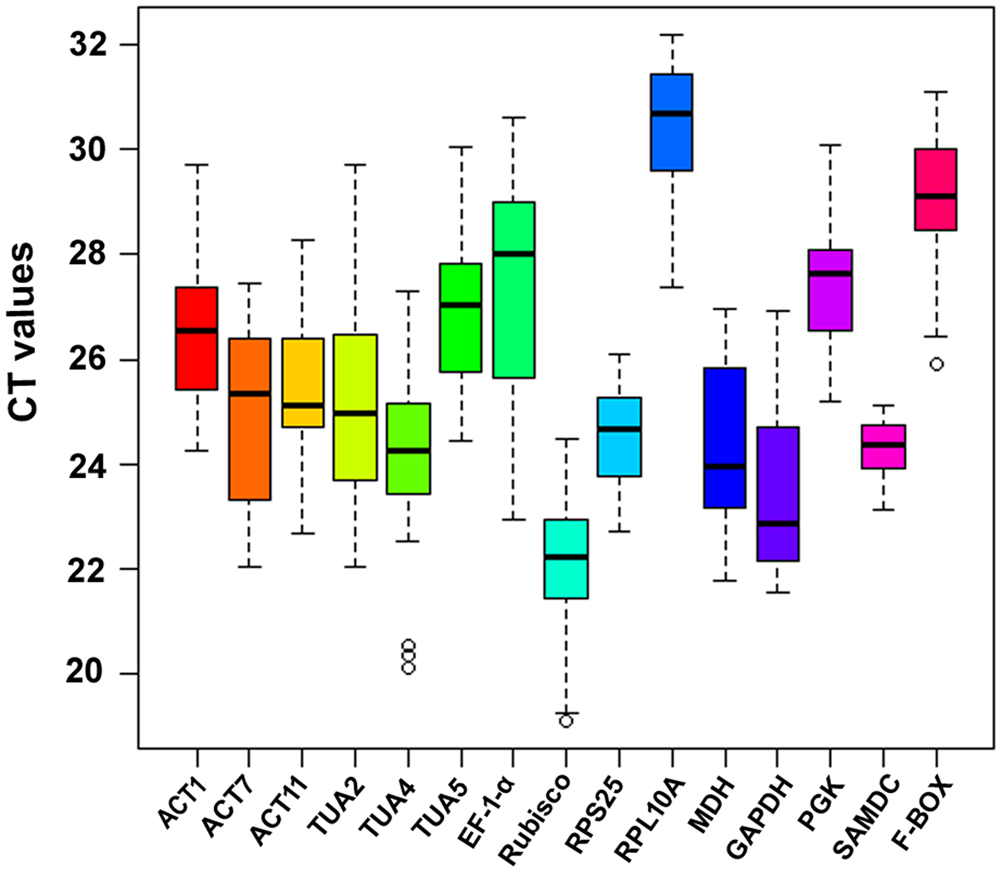
Distribution of threshold cycle (CT) values of 15 candidate reference genes across all 22 samples. The solid line within each box represents the 50th percentile. The lower boundary and upper boundary of each box represents the 25th and 75th percentile, respectively. The circles represent potential outliers.

The expression levels of the candidate reference genes were determined by qRT-PCR across 5 subsets of samples including different tissues, developmental stages, photosynthetic cycles, cold and drought treatments. The candidate reference genes displayed wide range of accumulation level across all the tested samples, with threshold cycle (CT) values spanning 19.48–31.79 (Fig. 1). Among them, *Rubisco* exhibited the highest expression abundance, with median CT value of 21.93. Both *GAPDH* and *MDH* showed relative high expression, with median CT values of 22.44 and 23.69, respectively. In contrast, *RPL10A* expressed lowest, with a median CT value of 30.38 (Fig. 1). Additionally, candidate genes showed distinct expression variability, among which *SAMDC* and *RPS25* displayed relative narrower CT range values than others (Fig. 1), showing that these genes expressed more stably. Importantly, however, these results revealed that none of the selected reference genes were expressed constantly in all samples tested from *N*. *cadamba*. Hence, it was necessary to validate suitable reference genes for normalization under different experimental conditions in *N*. *cadamba*.

### Expression stability of candidate reference genes

#### geNorm analysis

**Figure 2:**
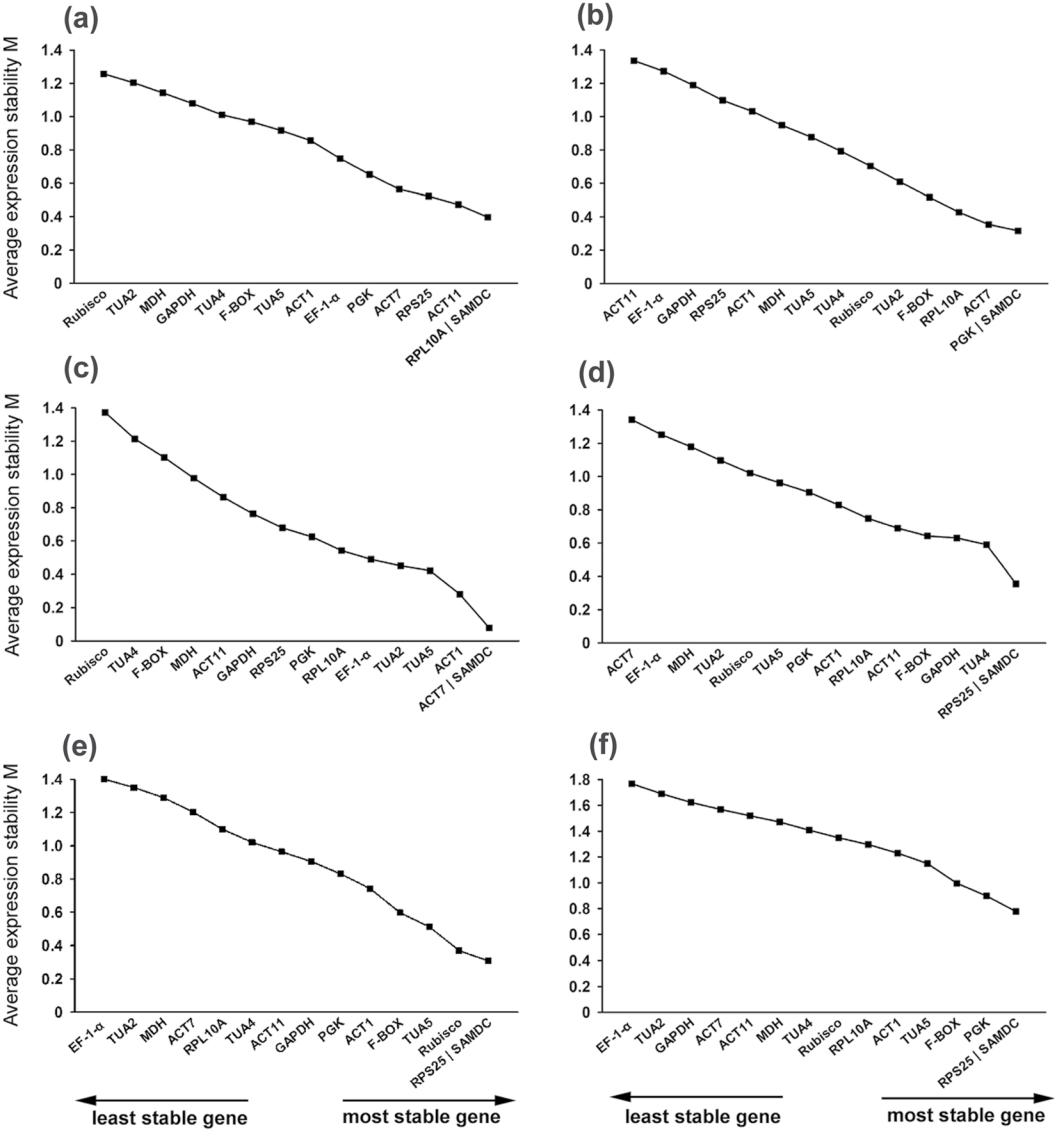
Average expression stability values (M) of 15 candidate reference genes calculated by geNorm. (**a**) Tissue; (**b**) developmental stage; (**c**) photosynthetic cycles; (**d**) drought treatment; (**e**) cold treatment; (**f)** all of the samples in our given conditions.

In order to rank the candidate reference genes under tested conditions, four commonly used gene expression analysis software programs, i.e., geNorm, NormFinder, BestKeeper, and RefFinder were applied to assess the expression stabilities of fifteen candidate reference genes. In geNorm analysis, the expression stability measure M was calculated for each gene based on the non-normalized expression level Q. A cut-off value of 1.5 was recommended and set for M to evaluate gene stability. For each sample groups in our study, M value in all the candidate genes was lower than 1.5 (Fig. 2a–e). All fifteen candidate genes in each sample group set were ranked by M value. Base on this analysis, a lower M value represents a higher degree of expression stability for the selected reference gene. For tissue group, *RPL10A* and *SAMDC* were the top ranked candidates, followed by *ACT11* and *RPS25* (Fig. 2a). *SAMDC*, *PGK*, *ACT7* and *RPL10A* were the four most stable reference genes in the mature leaves at different developmental stage (Fig. 2b). Among the samples of different photosynthetic cycles in a day, *ACT7*, *SAMDC* and *ACT1* exhibited the three most stable reference genes (Fig. 2c). *SAMDC* and *RPS25* with M value of about 0.3 ranked as the most stable genes in both groups of drought and cold treatment (Fig. 2d–e). When all samples were mixed and tested, most of the candidate genes had higher M values than their respective calculation in each group, four of which including *EF-1-α*, *TUA2*, *GAPDH* and *ACT7* even excessed the cut-off value of 1.5. *SAMDC* and *RPS25* were the two most stable candidates with M value of 0.78 (Fig. 2f).

**Figure 3:**
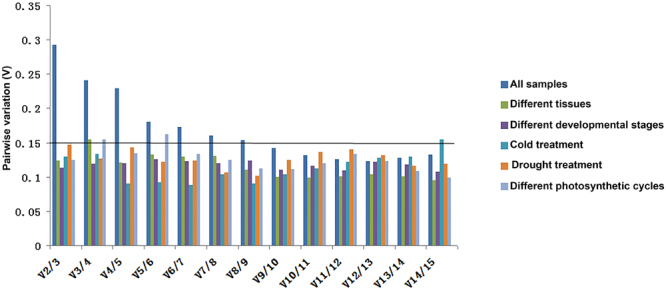
Determination of the optimal number of reference genes for normalization in the tested experimental conditions. geNorm was used to calculate the normalization factor (NF) from at least two genes; the variable V defines the pair-wise variation between two sequential NF values.

In certain conditions, multiple references might be more reliable than a single one for normalization in qRT-PCR assay^1^. To determine the minimum number of reference genes for accurate normalization, GeNorm was applied through calculating the pairwise variation (Vn/n + 1) between two sequential normalization factors to determine whether additional reference gene is necessary. Utilizing this software program, 0.15 was set as a cut-off for V value to determine whether additional reference genes be required^1^. In our study, for each sample group, a pairwise variation value of V2/3 was lower than 0.15 (Fig. 3), suggesting that only two reference genes were sufficient for normalization by qRT-PCR. Specifically, combination of *RPL10A* and *SAMDC* were the optimal reference genes for different tissues, while *PGK* and *SAMDC* were optimal for mature leaves at distinct development stages. Interestingly, combination of *RPS25* and *SAMDC* were the best choice for normalization under both drought and cold stress conditions, whereas *ACT7* and *SAMDC* were the best combination for samples under different photosynthetic cycles. However, when all the samples were analyzed simultaneously, nine reference genes were required since V8/9 was higher than 0.15 whereas V9/10 was below the cut-off value (Fig. 3). This suggested that no reasonable number of reference genes would be suitable for comparing expression levels between large numbers of tissues/growth conditions since the requirement to use nine references to calculate gene expression by qRT-PCR would be unreasonably time consuming. Alternatively, it may have been possible that a pairwise analysis cut-off value of 0.15 was too strict for this case, and this result also suggested that it is hard to find out universal reference genes for samples under different experimental conditions. Despite this limitation, we were able to identify suitable reference genes that exhibited specificity under a certain conditions.

### NormFinder analysis

**Figure 4:**
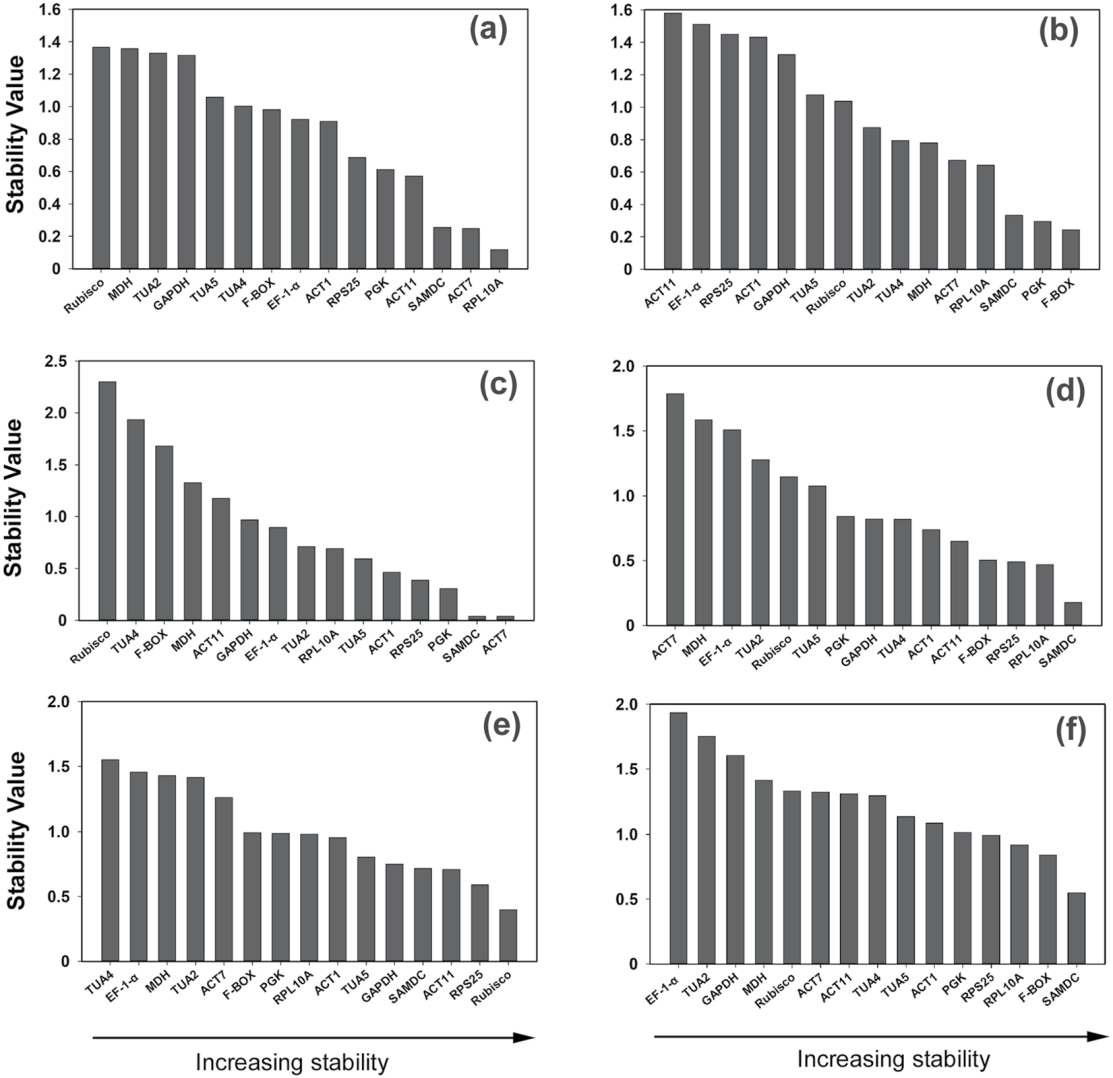
The stability value of 15 candidate reference genes calculated by NormFinder. The lower value indicated the higher stability of gene expression. **(a**) Tissue; (**b**) developmental stage; (**c**) photosynthetic cycles; (**d**) drought treatment; (**e**) cold treatment; (**f**) all of the samples in our given conditions.

To further confirm the stability of the reference genes obtained by the geNorm software, we applied the NormFinder software for optimal normalization genes identification among the candidates. The statistic algorithm of NormFinder is different from geNorm, the former considers intra- and intergroup variations for the calculation of normalization factors (NF), which were used to estimate the stability values of each reference gene. For the sample group of different tissues, *RPL10A*, *ACT7*, *SAMDC* and *ACT11* were the four top ranked candidates (Fig. 4a). Five stable references including *F-BOX*, *PGK*, *SAMDC*, *RPL10A*, and *ACT7* were top ranked in the sample set of mature leaves at different development stages, which showed good agreement with geNorm analysis but with slight changes in the ranking order (Fig. 4b). For the sample set of different photosynthetic cycle, *ACT7* and *SAMDC* were the most stable candidates (Fig. 4c). *SAMDC* was the most suitable references under drought stress (Fig. 4d), whereas it was ranked second according to geNorm. Consistent with geNorm analysis, *Rubisco* and *RPS25* were the best candidates for normalization under cold condition (Fig. 4e). For all the sample groups, the three top ranked references were *SAMDC*, *F-BOX* and *RPL10A*, of which *SAMDC* was the best single candidate with stability value of 0.548.

### BestKeeper analysis

The expression stability of the candidate reference genes were reanalyzed by another program BestKeeper. CT value and efficiencies of each primer were used as input data to calculate the standard deviation (SD), in which lower SD suggested higher stability of reference gene. *ACT7*, which had the lowest SD value of 0.36, was the most stable candidate in the sample group of different tissues, whereas *MDH* was the least stable gene with the highest SD (Table 1). *PGK*, *RPL10A* and *SAMDC* had SD value of less than 0.6, which were the three top ranked references in the sample set of mature leaves at different developmental stage. *SAMDC* were the most stable gene in the sample set of different photosynthetic cycles. Similarly, for the abiotic stress, *SAMDC* was the most suitable reference gene, followed by *RPS25* and *RPL10A* under both cold and drought conditions, respectively. When considering all samples in our study, *F-BOX* and *SAMDC* were the top two ranked reference genes.

**Table 1:** Expression stability values for *Neolamarckia cadamba* candidate reference genes calculated by BestKeeper.

Ranking	Tissue	Developmental stage	photosynthetic cycle	Drought treatment	Cold treatment	Total
1	*ACT7* (1.37 ± 0.36)	*PGK* (1.83 ± 0.51)	*SAMDC* (0.35 ± 0.09)	*RPL10A* (0.61 ± 0.19)	*RPS25* (0.67 ± 0.17)	*SAMDC* (1.43 ± 0.35)
2	*RPL10A* (1.43 ± 0.45)	*RPL10A* (1.91 ± 0.58)	*ACT7* (0.64 ± 0.14)	*SAMDC* (1.04 ± 0.25)	*SAMDC* (1.35 ± 0.33)	*F-BOX* (2.71 ± 0.79)
3	*SAMDC* (1.53 ± 0.37)	*SAMDC* (2.1 ± 0.51)	*PGK* (1.32 ± 0.37)	*RPS25* (1.38 ± 0.33)	*TUA5* (1.81 ± 0.51)	*RPS25* (2.78 ± 0.68)
4	*EF-1-α* (1.96 ± 0.58)	*F-BOX* (2.67 ± 0.78)	*RPS25* (1.57 ± 0.39)	*ACT1* (1.41 ± 0.38)	*Rubisco* (1.87 ± 0.41)	*PGK* (2.85 ± 0.78)
5	*PGK* (2 ± 0.56)	*ACT7* (2.79 ± 0.68)	*ACT1* (1.6 ± 0.41)	*ACT11* (1.75 ± 0.46)	*RPL10A* (2.6 ± A0.79)	*RPL10A* (3.33 ± 1.01)
6	*F-BOX* (2.11 ± 0.62)	*TUA5* (2.94 ± 0.75)	*TUA5* (1.6 ± 0.41)	*F-BOX* (1.79 ± 0.52)	*ACT11* (2.64 ± 0.64)	*TUA5* (3.58 ± 0.96)
7	*TUA5* (2.17 ± 0.6)	*TUA2* (3.09 ± 0.76)	*RPL10A* (1.94 ± 0.55)	*TUA5* (1.97 ± 0.53)	*F-BOX* (2.94 ± 0.87)	*ACT11* (3.86 ± 0.98)
8	*ACT11* (2.26 ± 0.57)	*RPS25* (3.09 ± 0.76)	*TUA2* (2.13 ± 0.49)	*TUA4* (2.37 ± 0.59)	*TUA4* (3.1 ± 0.77)	*ACT1* (4 ± 1.06)
9	*RPS25* (2.46 ± 0.6)	*MDH* (3.45 ± 0.83)	*EF-1-α* (2.54 ± 0.61)	*PGK* (2.52 ± 0.67)	*ACT1* (3.26 ± 0.88)	*TUA4* (4.41 ± 1.07)
10	*ACT1* (2.54 ± 0.66)	*TUA4* (3.52 ± 0.86)	*ACT11* (3.11 ± 0.79)	*MDH* (3.76 ± 0.91)	*PGK* (3.61 ± 0.99)	*MDH* (4.94 ± 1.2)
11	*TUA4* (3.22 ± 0.77)	*EF-1-α* (3.97 ± 1.11)	*GAPDH* (3.17 ± 0.8)	*TUA2* (4.97 ± 1.36)	*ACT7* (3.76 ± 0.95)	*Rubisco* (5.04 ± 1.1)
12	*TUA2* (4.48 ± 1.09)	*ACT11* (4.46 ± 1.17)	*MDH* (4.11 ± 1.02)	*EF-1-α* (5.12 ± 1.37)	*EF-1-α* (3.84 ± 1.06)	*ACT7* (5.26 ± 1.32)
13	*GAPDH* (4.69 ± 1.09)	*ACT1* (4.74 ± 1.3)	*F-BOX* (4.28 ± 1.21)	*ACT7* (5.33 ± 1.36)	*GAPDH* (4.44 ± 1.02)	*GAPDH* (5.77 ± 1.35)
14	*Rubisco* (5.2 ± 1.16)	*Rubisco* (5.18 ± 1.13)	*TUA4* (5.89 ± 1.31)	*Rubisco* (6.16 ± 1.32)	*TUA2* (5.07 ± 1.32)	*TUA2* (6.21 ± 1.57)
15	*MDH* (5.28 ± 1.24)	*GAPDH* (5.31 ± 1.21)	*Rubisco* (7.17 ± 1.56)	*GAPDH* (6.23 ± 1.48)	*MDH* (6.25 ± 1.56)	*EF-1-α* (6.44 ± 1.77)

Note: Fifteen candidate reference genes are evaluated by the lowest values of the coefficient of variance (CV) and standard deviation (SD). The number in the bracket indicated that CV ± SD.

### RefFinder analysis

The three different programs used for stability analysis showed different ranking orders of candidate reference genes. Therefore, to obtain a consensus result, we used RefFinder program to reorder the selected reference genes comprehensively (Table 2), and the integrated comparison of the evaluation by four programs was shown in Supplementary Table S2. The ranking order by RefFinder showed that the top two ranked reference gene were in agreement with the list yielded by geNorm under all given experimental conditions. *Rubisco* was ranked at the bottom for the group of different tissues and photosynthetic cycle; *EF-1-α* was at the bottom position for cold treatment and all samples tested. *ACT11* and *ACT7* were the least stable genes in the samples of different developmental stage and drought stress, respectively.

**Table 2:** Comprehensive ranking of 15 candidate reference genes integrated by RefFinder.

Ranking	Tissue	Developmental stage	Photosynthetic cycle	Drought treatment	Cold treatment	Total
1	*RPL10A*	*PGK*	*SAMDC*	*SAMDC*	*RPS25*	*SAMDC*
2	*SAMDC*	*SAMDC*	*ACT7*	*RPS25*	*SAMDC*	*RPS25*
3	*ACT7*	*F-BOX*	*ACT1*	*RPL10A*	*Rubisco*	*F-BOX*
4	*ACT11*	*RPL10A*	*PGK*	*F-BOX*	*TUA5*	*PGK*
5	*PGK*	*ACT7*	*TUA5*	*ACT11*	*ACT11*	*RPL10A*
6	*RPS25*	*TUA2*	*RPS25*	*ACT1*	*F-BOX*	*TUA5*
7	*EF-1-α*	*MDH*	*TUA2*	*TUA4*	*GAPDH*	*ACT1*
8	*ACT1*	*TUA4*	*RPL10A*	*GAPDH*	*ACT1*	*TUA4*
9	*F-BOX*	*TUA5*	*EF-1-α*	*TUA5*	*RPL10A*	*ACT11*
10	*TUA5*	*Rubisco*	*GAPDH*	*PGK*	*PGK*	*Rubisco*
11	*TUA4*	*RPS25*	*ACT11*	*Rubisco*	*TUA4*	*MDH*
12	*GAPDH*	*GAPDH*	*MDH*	*TUA2*	*ACT7*	*ACT7*
13	*TUA2*	*ACT1*	*F-BOX*	*MDH*	*TUA2*	*GAPDH*
14	*MDH*	*EF-1-α*	*TUA4*	*EF-1-α*	*MDH*	*TUA2*
15	*Rubisco*	*ACT11*	*Rubisco*	*ACT7*	*EF-1-α*	*EF-1-α*

### Validation of reference genes

**Figure 5:**
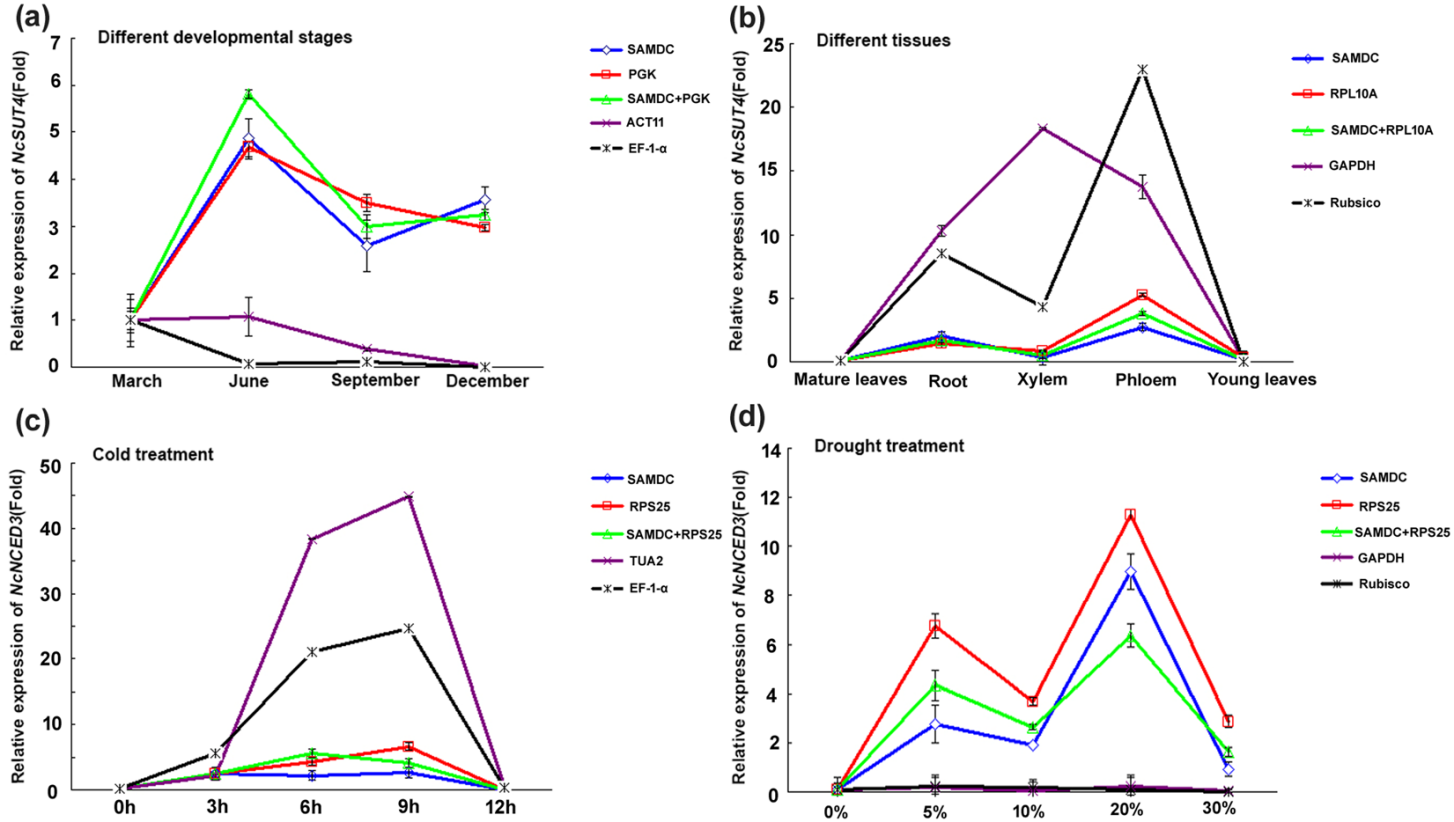
Relative quantification of *NcSUT4* and *NcNCED3* expression levels using the most and least stable reference genes for normalization in the given experimental conditions. (**a**) Expression level of *NcSUT4* at different developmental stages. Most stable reference genes (*SAMDC*, *PGK*) and least stable reference genes (*ACT11*, *EF-1-α*) were used, respectively; (**b**) expression level of *NcSUT4* in different tissues. Most stable reference genes (*SAMDC*, *RPL10A*) and least stable reference genes (*GAPDH*, *Rubisco*) were used, respectively; (**c**) expression level of *NcNCED3* under cold treatment. Most stable reference genes (*SAMDC*, *RPS25*) and least stable reference genes (*TUA2*, *EF-1-α*) were used, respectively; (**d**) expression level of *NcNCED3* under drought treatment. Most stable reference genes (*SAMDC*, *RPS25*) and least stable reference genes (*GAPDH*, *Rubisco*) recommended by BestKeeper were used, respectively. The error bars represent the mean of three biological replicates ± SD.

In order to verify the stability of reference gene in this study, we analyzed the expression levels of sucrose transporter 4 (*NcSUT4*) and 9‐cis‐epoxycarotenoid dioxygenase 3 (*NcNCED3*) using the selected reference genes. Sucrose transporters are responsible for phloem loading, transport and unloading of sucrose from source to sink^36^. *NCED* genes encode key enzymes for the synthesis of ABA, and are induced under drought stress^37,38^. The transcript levels of *NcSUT4* were quantified in the samples of different developmental stages and different tissues, using two stable reference genes and two unstable reference genes as endogenous controls, respectively (Fig. 5a,b). As shown in Fig. 5a, when using the two stable reference genes (*SAMDC* or *PGK*) for normalization, the expression level of *NcSUT4* was increased in the mature leaves from March to December, with high peak in June. However, when using the least stable reference genes *ACT7* and *EF-1-α* as normalization factors, *NcSUT4* exhibited totally different expression pattern, with gradually decrease across different development stages of mature leaves. In various tissues, relative higher transcript abundance of *NcSUT4* was found in root and phloem when using the two most stable reference genes (*SAMDC* and *RPL10A*) singly or in combination. Though the expression pattern was similar when the unstable gene *Rubisco* served as internal control, the relative expression of *NcSUT4* was much higher in root, xylem and phloem than that using the most stable references. In contrast, the highest expression of *NcSUT4* was found in xylem rather than phloem when using another unstable endogenous gene *GAPDH* (Fig. 5b). The relative transcript abundance of *NcNCED3* under cold and drought stress condition was also calculated in the way similar with *NcSUT4* (Fig. 5c,d). Under cold treatment, the expression level of *NcNCED3* was not affected significantly when normalized by the most stable reference genes *SAMD*C and *RPS25*, whereas the transcript abundance was overestimated when using the least stable references *TUA2* and *EF-1-α*. With drought treatment, the relative expression level of *NcNCED3* increased to two peaks at the PEG concentration of 5% and 20%. On the contrary, normalization by the two least stable genes (*GAPDH*, *Rubisco*) led to the nearly unchanged pattern of *NcNCED3* expression level. Taken together, the substantial divergence was found in the expression levels of *NcSUT4* and *NcNCED3* when normalized by the most stable and least stable reference genes.

## Discussion

Gene expression analysis has become an important aspect in the functional investigation of genes during the growth and development of various living organisms. The accuracy of genes relative expression mainly depends on the reference genes. Therefore, the selection of inappropriate reference gene can give rise to the erroneous data and misinterpretation of experimental results^39^. An ideal reference gene for normalization in qRT-PCR analysis should be stably expressed at moderate level in a variety of test samples and has similar amplification efficiency to target genes^25,40^. However, in our analysis of potential candidate reference genes in *Neolamarckia cadamba*, we determined that there is no single reference gene that exhibited constant expression level in all the samples of various tissues and under different experimental conditions, similar with the findings in cotton^41^. Even though housekeeping genes have been commonly used for normalization in qRT-PCR assay, the expression levels of several genes like *ACT*, *18S rRNA* and *GAPDH* were not always constant in the given conditions. Therefore, it is necessary to assess the expression stability of candidate reference genes systematically when performing qRT-PCR analysis for different tissue and experimental conditions.

In this study, 15 candidate genes were selected for identifying the best reference among 22 samples under different experimental conditions and a variety of various tissues from *N*. *cadamba*. By the use of four computational gene expression analysis programs, i.e. geNorm, NormFinder, BestKeeper and RefFinder, the expression stability of the selected candidate reference genes were evaluated. The statistical algorithms to assess the stability of references of three programs vary greatly. geNorm estimates the average pairwise variation of a reference gene versus all other genes among the given samples^1,11^. However, for NormFinder analysis, the expression stability value was calculated based on intra- and inter-group variation^19^. In BestKeeper, the CV and SD values determine the stability ranking of a candidate reference gene^20^. Owing to the different algorithms of the three software programs used in this study, the ranking order of the selected candidates showed variable degrees of agreement (Figs 2, 4, Table 1 and Supplementary Table S2). The reference genes, *SAMDC* exhibited the most stable expression based on analysis by all three programs across all 22 samples under the five given conditions (see Supplementary Table S2). In addition, *RPL10A* was one of top two most stable reference genes in various tissues by all three programs. However, *PGK*, one top ranked gene by geNorm and BestKeeper in the sample group of different developmental stages, was ranked at a medium position in NormFinder (Fig. 4). Similarly, under cold stress condition, expression of *RPS25* was the least stable according to NormFinder, whereas it was the most stable gene by geNorm and BestKeeper. Increasing evidences have demonstrated that two or three algorithms were widely used to evaluate expression stability of the reference genes for normalizing mRNA and miRNA expression^5,24,41,42^. Specially, we also noticed that the unstable reference genes identified by three programs were similar. For example, *TUA2*, *GAPDH*, *Rubisco* and *MDH* were expressed unstably in different tissues by all three algorithms. Furthermore, a web-based tool RefFinder was employed to integrate and generate the comprehensive ranking of the candidate reference genes based on the geometric mean of the weights of every gene calculating by each program^43^. The result produced by RefFinder provides us the overall ranking order, which has been widely used in the previous studies on exploring the suitable reference genes in the certain conditions^7,9,18,24^.

Our study showed that *SAMDC* was the top ranked gene when all samples were analyzed, since it was recommended by all three programs as the most stable reference gene (Figs 2, 4, and Table 2). Interestingly, the integrated ranking order generated by RefFinder suggested that *SAMDC* was also the optimal reference gene in five independent sample sets, including the samples under cold and drought stresses, which was similar with the result of *Brachypodium distachyon* plant grown under various stresses conditions^44^. *SAMDC*, encoding S-adenosylmethionine decarboxylase, is a rate-limiting enzyme in the biosynthesis of the polyamines spermidine and spermine^45,46^. *SAMDC* expressed ubiquitously in different plant organs in *Brachypodium*^44^, and highly induced under various abiotic stress treatments in rice^47^, indicating it might play essential roles in plant growth and tolerance response. *SAMDC* has never been used as internal reference gene for normalization *N*. *cadamba*, and thereby would be considered as a novel reference candidate. However, the superior stability performance of *SAMDC* was not found in some species under certain conditions. For example, *SAMDC* was considered one of the least stable reference genes in orchardgrass under various abiotic conditions and different tissues of banana^5,7^. In addition to *SAMDC*, *PGK* was also found to be the best stable candidate as reference gene in the leaves at different developmental stages in our study. PGK, encoding phosphoglycerate kinase, plays pivotal roles in the glycolytic pathway^48^. *PGK* was found to be one of the superior reference genes for normalization in chrysanthemum of cross-ploidy levels and in tomato leaves during light stress^49,50^, suggesting that *PGK* had potential to be a suitable reference in certain experimental conditions., Likewise, our current study showed that *PGK* was the most stable for different stages, which will be a new reference candidate in *N*. *cadamba*.

Previous reports have demonstrated that using multiple reference genes rather than a single one for was more robust and accurate for normalizing qRT-PCR data^1,49^. Accordingly, more and more studies in various species were tended to apply multiple references^51–53^. In our study, the combination of two reference candidates was sufficient for normalization in the five different experimental sets (Fig. 2). Thus, additional genes were not required for gene expression normalization. However, it was not always to use multiple reference genes due to the cost, and also it would be dependent on the variability of the selected reference genes.

The housekeeping genes have long been used as internal control to quantify gene expression for their requirement in maintaining the basal cellular functions. They were supposed to be highly conserved and stably expressed under different experimental conditions and various developmental stages^15,54^. *ACTIN*, *GAPDH*, *TUB* and *EF*, considered as classical housekeeping genes, have been commonly used as reference genes in qRT-PCR analysis in the model plants, as well as the non-model species^55–58^. Unfortunately, increasing studies demonstrated the housekeeping genes would variably expressed in many species under given experimental sets, which would not be suitable to serve as reference genes for normalization. *ACT* and *GAPDH*, for instance, were not appropriate candidate reference under many experimental conditions in papaya^18^, similar with the result of chicory in seedlings and cell cultures^42^. In the present study, three *ACT* genes were chosen to evaluate their stabilities in 22 samples from *N*. *cadamba*. When all samples were tested, *ACT7* was ranked at the bottom position while *ACT1* was ranked intermediate position, contrast with the result in samples at different developmental stages with top-ranked *ACT7* and bottom-ranked *ACT1* (Table 2). For the separate sample groups, *ACT7* and *ACT1* ranked among the top three most stable reference genes under different photosynthesis cycle, while *ACT7* had poor performance under drought stress condition. Liu *et al*.^26^ have proposed that genes with similar function might have various performances in expression stability. In our work, the ribosomal protein *RPS25* and *RPL10A* were considered as the appropriate reference genes when all sample were tested, whereas they performed variably with top ranking in *RPL10A* and middle-bottom ranking in *RPS25*. Another classical housekeeping gene *TUA*, encoding one of the major components of microtubules^59^, had a moderate or least stable level in most of our given experimental sets, but considered as the most stable candidate in poplar^60^ and least stable reference in *Caragana korshinskii* Kom.^61^. These results further confirmed that there is no universal reference gene across all species under various experimental conditions. Accordingly, stability assessment of the traditional reference genes is necessary prior to use in qRT-PCR.

To verify the suitability of the reference genes identified in this study, the relative expression levels of *NcSUT4* and *NcNCED3* under different experimental conditions have been detected. The expression profile of *NcSUT4* and *NcNCED3* were normalized by best suitable genes as well as least unstable genes recommended by RefFinder in their own experimental sets. The results revealed that expression pattern of *NcSUT4* and *NcNCED3* were obviously over- or underestimated when using the unstable genes for normalization (Fig. 5), suggesting that selection of appropriate stable reference genes is critically important for the correct normalization for qRT-PCR data in *N*. *cadamba*.

In conclusion, our study presented the systematic evaluation of a set of candidate reference genes as normalization factors in qRT-PCR analysis in the samples under a wide range of experimental conditions in *N*. *cadamba*. Four widely used programs (geNorm, NormFinder, BestKeeper and RefFinder) were applied to estimate the expression stability of the selected candidate genes. The results of comprehensive ranking order showed that *SAMDC* displayed highest stability across the set of all samples, mature leaves at different photosynthetic cycles and under drought stress, whereas *RPL10A* had the most stable expression in various tissues. *PGK* and *RPS25* were the most stable in mature leaves at different developmental stages and under cold conditions, respectively. The expression analysis of *NcSUT4* and *NcNCED3* emphasized the importance of suitable reference gene to get accurate and reliable quantitation results by qRT-PCR. For the first time in *N*. *cadamba*, our study provided the optimal reference under different experimental conditions, which would be of great importance in further analysis of gene expression and facilitate the understanding of underlying mechanisms in the aspects of development and stress response.

## Materials and Methods

### Plant materials

Fresh young leaves, mature leaves, phloem, xylem, roots of 4–5 year-old *Neolamarckia cadamba* from South China Agricultural University Yuejin North experimental field were harvested. For samples of developmental stages, mature leaves were collected on March, June, September and December. For samples of different photosynthetic cycle, mature leaves were sampled at 7:00, 13:00 and 19:00 on October 28, 2016. For drought treatment samples, the seedlings after seven days of transplantation from tissue culture were treated with 0%, 5%, 10%, 20% and 30% concentration of PEG 6000 and the mature leaves were collected. Meanwhile, the seedlings were also transferred to 4 °C for chilling stress, and the mature leaves were taken after 0, 3, 6, 9 and 12 h. Each sample was collected with three biological replicates. Information of all five sample sets mentioned above is summarized in Supplementary Table S3. Samples were frozen immediately in liquid nitrogen and stored at −80 °C until RNA extraction.

### RNA isolation, quality control and cDNA synthesis

Total RNA was extracted from the samples using OmniPlant RNA Kit (CWBIO) with DNase I treatment to avoid genomic DNA containment. The RNA purity and integrity were estimated by calculating their A260/280 and A260/A230 absorbance ratios and agarose gel electrophoresis analysis. cDNA was synthesized from 0.5 μg of total RNA by Hiscript II Reverse Transcriptase kit (Vazyme) according to the manufacture’s instruction. The cDNA were diluted 30-fold for subsequent qRT-PCR analysis.

### Selection of candidate reference gene and primers design

Fifteen candidate reference genes, including Actin 1 (ACT1), Actin 7 (ACT7), Actin 11 (ACT11), Tubulin alpha 2 (TUA2), Tubulin alpha 4 (TUA4), Tubulin alpha 5 (TUA5), Elongation factor 1-alpha (EF-1-α), Ribulose bisphosphate carboxylase (Rubisco), Ribosomal protein S25 (RPS25), Ribosomal protein L10A (RPL10A), Malate dehydrogenase (MDH), Glyceraldehyde-3-phosphate dehydrogenase (GAPDH), Phosphoglycerate kinase (PGK), S-adenosylmethionine decarboxylase (SAMDC), F-Box protein (F-BOX) were selected, based on a preliminary *in silico* evaluation of gene expression stability using *in house* RNA-Seq libraries from various tissues of *N*. *cadamba* including leaves, xylem, cambium and phloem (unpublished data). Although gene expression study is common on plant functional genomic research, *N*. *cadamba* is still a less studied species. To date, only two studies on gene expression have been carried out in *N*. *cadamba*, and cyclophilin was used as the internal reference for normalization^31,33^. However, according to the transcriptome data of leaves, xylem, cambium and phloem, the expression of cyclophilin varied greatly in these samples (unpublished data). Therefore, cyclophilin was not selected for further evaluation of expression stability in our study. Due to lacking the genome sequence of *N*. *cadamba*, the primers were designed based on sequences extracted from the released transcriptome data^31^. Primers for qRT-PCR were designed using web-based Primer-BLAST tool in NCBI (https://www.ncbi.nlm.nih.gov/tools/primer-blast/) with default parameters. Gene ID, primers and the expected length of each gene were indicated in Supplementary Table S4. All the primers were designed across introns except *F-BOX* and *RPL10A*. To check the specificity of the primers, general PCR was carried out and the products was verified by electrophoresis on 1% agarose gels.

### Quantitative Real-time PCR

qRT-PCR was performed on LightCycler480 (Roche Molecular Biochemicals, Mannheim, Germany) with optical 96-well plate. 10 μL of the reaction mix containing 5 μL AceQ qPCR SYBR GREEN PCR Master Mix, 0.5 μL diluted cDNA template and 0.5 μL each primer were added into each well. The thermal cycling profile was recommended by the manufacture: 95 °C for 10 min, 40 cycles of 95 °C for 15 s, 60 °C for 30 s. To confirm the specificity of the primers, melting curves were included after amplification. All the samples for qRT-PCR analysis were conducted with three biological replicates each comprising three technical replicates. To calculate the gene-specific PCR efficiency (E) and correlation coefficient (R^2^) for each gene, standard curves were generated using tenfold dilution series from the mixed cDNA.

### Analysis of the stabilities of reference genes

Three software tools including geNorm^1^ (version 3.5, http://medgen.ugent.be/*jvdesomp/genorm/), NormFinder^19^ (http://www.mdl.dk/publicationsnormfinder.htm) and BestKeeper^20^ (http://www.gene-quantification.de/bestkeeper.html) were used to evaluate the stability of the 15 selected candidate reference genes across all the experimental sets. Expression levels of the candidate reference genes were determined by cycle threshold (CT) values. All the procedures of statistical analyses by the above packages were conducted according to the manufacturer’s instructions.

The geNorm software calculates of gene expression stability value (M), which lower M value suggested higher gene’s expression stability. Moreover, geNorm also generated a pairwise stability measurement, which can be used to evaluate the suitable number of reference genes for normalization. NormFinder focuses on finding the two genes with the least intra- and inter-group expression variation or the most stable reference gene in intra-group expression variation. The principle of the BestKeeper program is to determine the stability of the reference gene by using the two parameters to calculate the correlation coefficient (CV) and the standard coefficient of variation (SD). Finally, the stability rankings of the reference genes from the three different algorithms were integrated, generating an overall ranking according to the geometric mean by RefFinder^43^.

### Validation of reference genes

To evaluated the reliability of the reference genes ranked by three algorithms, we analyzed the expression profiles of two target genes *9-Cis-epoxycarotenoid dioxygenase 3* (*NcNCED3*), and *Sucrose transporter 4* (*NcSUT4*) under the two most stable and two least stable reference genes. The expression level of *NcNCDE3* was determined under drought and cold treatment with forward primer TTTCGCGATAACTGAGAACT and reverse primer ACCAAACCTCGAAACTTTGT, while *NcSUT4* was quantified in various tissues and at different developmental stages of mature leaves with forward primer GGCTTTTGTTTTAGGGTTT and reverse primer CTCGAGTCCTCCTGTGATC. The E^−ΔΔCT^ method was used to calculate the relative expression levels^62^.

## Electronic supplementary material

Supplementary Information
