# Supplementary material for: Selection and Validation of Reference Genes for mRNA Expression by Quantitative Real-Time PCR Analysis in Neolamarckia cadamba
Source: Sci Rep. 2018 Jun 18;8:9311. doi: 10.1038/s41598-018-27633-5 (PMC6006177; doi:10.1038/s41598-018-27633-5)
Supplement: Supplementary file 1 — Supplementary Information [file 41598_2018_27633_MOESM1_ESM.pdf]

Supplementary Information

Selection and Validation of Reference Genes for mRNA Expression by Quantitative Real-Time PCR Analysis in *Neolamarckia cadamba*

Tian Huang [1, 2, §], Jian-Mei Long[2, §], Si-Wen Liu[1, 2], Zi-Wei Yang[1, 2], Qi-Jin Zhu[1, 2], Xiao-Lan Zhao[2], Chang-Cao Peng[1, 2, *]

1 State Key Laboratory for Conservation and Utilization of Subtropical Agro-bioresources, South China Agricultural University, Guangzhou 510642, China

2 Guangdong Key Laboratory for Innovative Development and Utilization of Forest Plant Germplasm, College of Forestry and Landscape Architecture, South China Agricultural University, Guangzhou 510642, China

§These authors contributed equally to this work.

Corresponding author

Corresponding author:

Chang-Cao Peng

Professor

State Key Laboratory for Conservation and Utilization of Subtropical Agro-bioresources, South China Agricultural University, Guangzhou 510642, China; Guangdong Key Laboratory for Innovative Development and Utilization of Forest Plant Germplasm, College of Forestry and Landscape Architecture, South China Agricultural University, Guangzhou 510642, China

Tel number: +86-20-85280259

E-mail: ccpeng@scau.edu.cn

Supplementary Information Description

Supplementary Figures

Supplementary Fig. S1 Specificity of primer pairs for qRT-qPCR amplification. 1.0% non-denaturing agarose gel electrophoresis showed amplification of a specific product of the expected size for each reference gene. M represented DNA marker. 1-15 represented *ACT1*, *ACT7*, *ACT11*, *TUA2*, *TUA4*, *TUA5*, *EF-1-α*, *Rubisco*, *RPS25*, *RPL10A*, *MDH*, *GAPDH*, *PGK*, *SAMDC*, *F-BOX*.

Supplementary Fig. S2 Melting curve of 15 candidate reference genes. a-o refered to *ACT1*, *ACT7*, *ACT11*, *TUA2*, *TUA4*, *TUA5*, *EF-1-α*, *Rubisco*, *RPS25*, *RPL10A*, *MDH*, *GAPDH*, *PGK*, *SAMDC* and *F-BOX*, respectively.

Supplementary Tables

Supplementary Table S1 Amplicon characteristics of candidate reference genes.

Supplementary Table S2 Stability ranking orders of candidates reference genes in six experimental sets by geNorm, NormFinder, BestKeeper and RefFinder.

Supplementary Table S3 Five sample sets from *Neolamarckia cadamba* used in this study.

Supplementary Table S4 Characteristics of candidate reference genes and qRT-PCR primers in *Neolamarckia cadamba*. F indicates forward primer while R represents reverse primer.

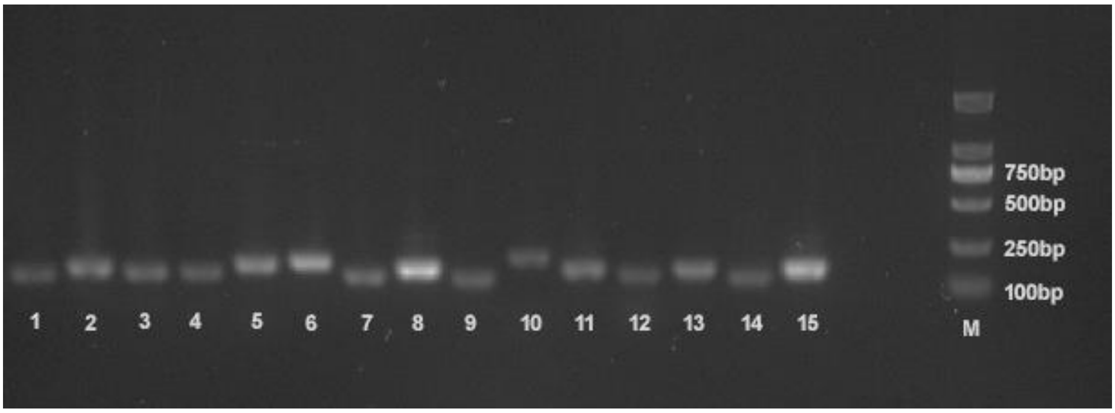

Supplementary Fig. S1 Specificity of primer pairs for qRT-qPCR amplification. 1.0% non-denaturing agarose gel electrophoresis showed amplification of a specific product of the expected size for each reference gene. M represented DNA marker. 1-15 represented *ACT1*, *ACT7*, *ACT11*, *TUA2*, *TUA4*, *TUA5*, *EF-1-α*, *Rubisco*, *RPS25*, *RPL10A*, *MDH*, *GAPDH*, *PGK*, *SAMDC*, *F-BOX*.

The raw image:

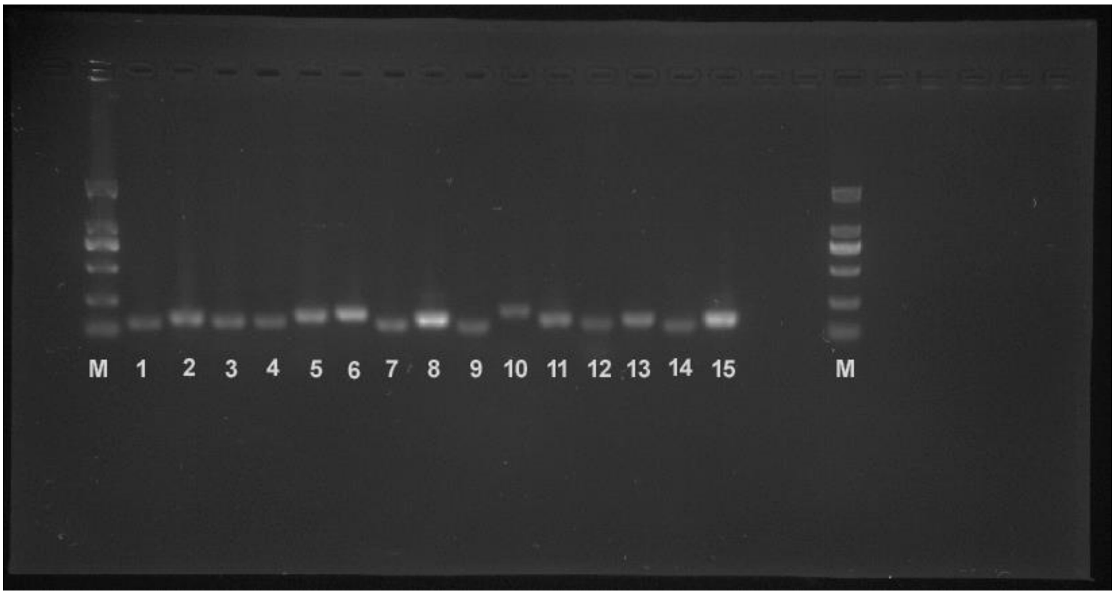

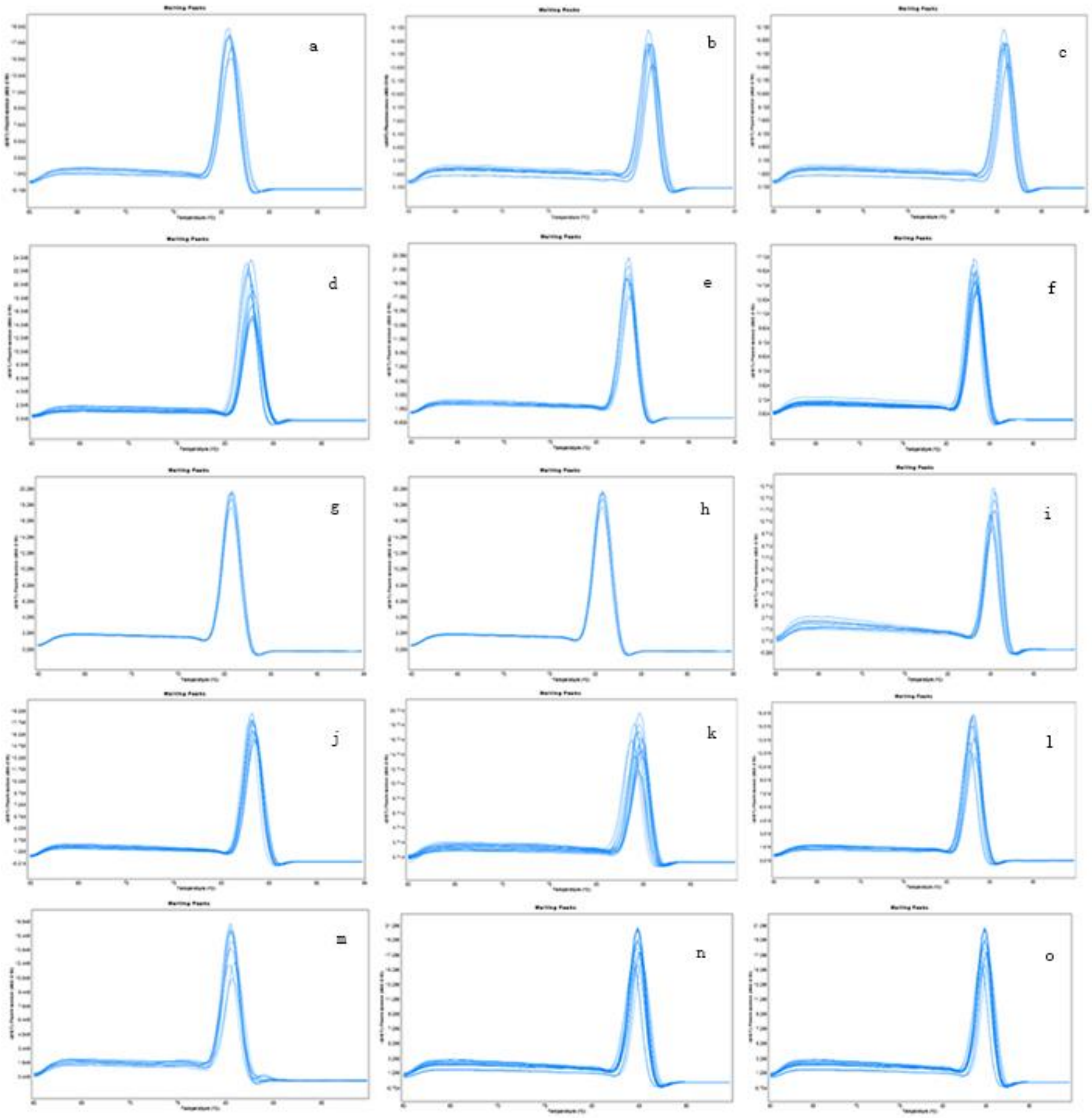

Supplementary Fig. S2 **Melting curve of 15 candidate reference genes.** a-o refered to *ACT1*, *ACT7*, *ACT11*, *TUA2*, *TUA4*, *TUA5*, *EF-1-α*, *Rubisco*, *RPS25*, *RPL10A*, *MDH*, *GAPDH*, *PGK*, *SAMDC* and *F-BOX*, respectively.

Supplementary Table S1 Amplicon characteristics of candidate reference genes.

Gene symbol	Linear regression equation	Correlation (R^2)	Amplification efficiency (%)	Slope
ACT1	y = -3.4600x + 31.185	0.9917	0.945	-3.4600
ACT7	y = -3.2903x + 33.513	0.9907	1.013	-3.2903
ACT11	y = -3.3053x + 32.799	0.9918	1.007	-3.3053
TUA2	y = -3.2273x + 32.080	0.9986	1.041	-3.2273
TUA4	y = -3.2273x + 32.080	0.9986	1.041	-3.2273
TUA5	y = -3.3399x + 32.758	0.9975	0.993	-3.3399
EF-1-α	y = -3.3950x + 31.979	0.9955	0.970	-3.3950
Rubisco	y = -3.2863x + 33.577	0.9902	1.015	-3.2863
RPS25	y = -2.9840x + 32.645	0.9991	1.163	-2.9840
RPL10A	y = -3.4037x + 33.979	0.9954	0.967	-3.4037
MDH	y = -3.1153x + 32.282	0.9944	1.094	-3.1153
GAPDH	y = -3.3027x + 34.511	0.9984	1.008	-3.3027
PGK	y = -3.2188x + 31.943	0.9953	1.045	-3.2188
SAMDC	y = -3.4283x + 31.231	0.9946	0.957	-3.4283
F-BOX	y = -3.1690x + 32.654	0.9936	1.068	-3.1690

Supplementary Table S2 Stability ranking orders of candidates reference genes in six experimental sets by geNorm, NormFinder, BestKeeper and RefFinder.

Method	Ranking order (The 1st is the most stable, and the 15th is the least stable)														
	**1**	**2**	**3**	**4**	**5**	**6**	**7**	**8**	**9**	**10**	**11**	**12**	**13**	**14**	**15**
(A) Ranking order in different tissues															
Genorm	*RPL10A* | *SAMDC*		*ACT11*	*RPS25*	*ACT7*	*PGK*	*EF-1-α*	*ACT1*	*TUA5*	*F-BOX*	*TUA4*	*GAPDH*	*MDH*	*TUA2*	*Rubisco*
Normfinder	*RPL10A*	*ACT7*	*SAMDC*	*ACT11*	*PGK*	*RPS25*	*ACT1*	*EF-1-α*	*F-BOX*	*TUA4*	*TUA5*	*GAPDH*	*TUA2*	*MDH*	*Rubisco*
BestKeeper	*ACT7*	*SAMDC*	*RPL10A*	*PGK*	*ACT11*	*EF-1-α*	*TUA5*	*RPS25*	*F-BOX*	*ACT1*	*TUA4*	*TUA2*	*GAPDH*	*Rubisco*	*MDH*
RefFinder	*RPL10A*	*SAMDC*	*ACT7*	*ACT11*	*PGK*	*RPS25*	*EF-1-α*	*ACT1*	*F-BOX*	*TUA5*	*TUA4*	*GAPDH*	*TUA2*	*MDH*	*Rubisco*
(B) Ranking order under different developmental stages															
Genorm	*PGK* | *SAMDC*		*ACT7*	*RPL10A*	*F-BOX*	*TUA2*	*Rubisco*	*TUA4*	*TUA5*	*MDH*	*ACT1*	*RPS25*	*GAPDH*	*EF-1-α*	*ACT11*
Normfinder	*F-BOX*	*PGK*	*SAMDC*	*RPL10A*	*ACT7*	*MDH*	*TUA4*	*TUA2*	*Rubisco*	*TUA5*	*GAPDH*	*ACT1*	*RPS25*	*EF-1-α*	*ACT11*
BestKeeper	*PGK*	*SAMDC*	*RPL10A*	*ACT7*	*TUA5*	*TUA2*	*RPS25*	*F-BOX*	*MDH*	*TUA4*	*EF-1-α*	*Rubisco*	*ACT11*	*GAPDH*	*ACT1*
RefFinder	*PGK*	*SAMDC*	*F-BOX*	*RPL10A*	*ACT7*	*TUA2*	*MDH*	*TUA4*	*TUA5*	*Rubisco*	*RPS25*	*GAPDH*	*ACT1*	*EF-1-α*	*ACT11*
(C) Ranking order under different photosynthetic cycles															
Genorm	*ACT7* | *SAMDC*		*ACT1*	*TUA5*	*TUA2*	*EF-1-α*	*RPL10A*	*PGK*	*RPS25*	*GAPDH*	*ACT11*	*MDH*	*F-BOX*	*TUA4*	*Rubisco*
Normfinder	*ACT7*	*SAMDC*	*PGK*	*RPS25*	*ACT1*	*TUA5*	*RPL10A*	*TUA2*	*EF-1-α*	*GAPDH*	*ACT11*	*MDH*	*F-BOX*	*TUA4*	*Rubisco*
BestKeeper	*SAMDC*	*ACT7*	*PGK*	*RPS25*	*ACT1*	*TUA5*	*TUA2*	*RPL10A*	*EF-1-α*	*ACT11*	*GAPDH*	*MDH*	*F-BOX*	*TUA4*	*Rubisco*
RefFinder	*SAMDC*	*ACT7*	*ACT1*	*PGK*	*TUA5*	*RPS25*	*TUA2*	*RPL10A*	*EF-1-α*	*GAPDH*	*ACT11*	*MDH*	*F-BOX*	*TUA4*	*Rubisco*
(D) Ranking order under drought stress															
Genorm	*RPS25* | *SAMDC*		*TUA4*	*GAPDH*	*F-BOX*	*ACT11*	*RPL10A*	*ACT1*	*PGK*	*TUA5*	*Rubisco*	*TUA2*	*MDH*	*EF-1-α*	*ACT7*
Normfinder	*SAMDC*	*RPL10A*	*RPS25*	*F-BOX*	*ACT11*	*ACT1*	*TUA4*	*GAPDH*	*PGK*	*TUA5*	*Rubisco*	*TUA2*	*EF-1-α*	*MDH*	*ACT7*
BestKeeper	*RPL10A*	*SAMDC*	*RPS25*	*ACT1*	*ACT11*	*F-BOX*	*TUA5*	*TUA4*	*PGK*	*MDH*	*Rubisco*	*TUA2*	*ACT7*	*EF-1-α*	*GAPDH*
RefFinder	*SAMDC*	*RPS25*	*RPL10A*	*F-BOX*	*ACT11*	*ACT1*	*TUA4*	*GAPDH*	*TUA5*	*PGK*	*Rubisco*	*TUA2*	*MDH*	*EF-1-α*	*ACT7*
(E) Ranking order under cold stress															
Genorm	*RPS25* | *SAMDC*		*Rubisco*	*TUA5*	*F-BOX*	*ACT1*	*PGK*	*GAPDH*	*ACT11*	*TUA4*	*RPL10A*	*ACT7*	*MDH*	*TUA2*	*EF-1-α*
Normfinder	*Rubisco*	*RPS25*	*ACT11*	*SAMDC*	*GAPDH*	*TUA5*	*ACT1*	*RPL10A*	*PGK*	*F-BOX*	*ACT7*	*TUA2*	*MDH*	*EF-1-α*	*TUA4*

Continued

BestKeeper	*RPS25*	*SAMDC*	*Rubisco*	*TUA5*	*ACT11*	*TUA4*	*RPL10A*	*F-BOX*	*ACT1*	*ACT7*	*PGK*	*GAPDH*	*EF-1-α*	*TUA2*	*MDH*
RefFinder	*RPS25*	*Rubisco*	*SAMDC*	*TUA5*	*ACT11*	*F-BOX*	*GAPDH*	*ACT1*	*RPL10A*	*PGK*	*TUA4*	*ACT7*	*TUA2*	*MDH*	*EF-1-α*
(F) Ranking order under all samples															
Genorm	*RPS25* | *SAMDC*		*PGK*	*F-BOX*	*TUA5*	*ACT1*	*RPL10A*	*Rubisco*	*TUA4*	*MDH*	*ACT11*	*ACT7*	*GAPDH*	*TUA2*	*EF-1-α*
Normfinder	*SAMDC*	*F-BOX*	*RPL10A*	*RPS25*	*PGK*	*ACT1*	*TUA5*	*TUA4*	*ACT11*	*ACT7*	*Rubisco*	*MDH*	*GAPDH*	*TUA2*	*EF-1-α*
BestKeeper	*SAMDC*	*RPS25*	*PGK*	*F-BOX*	*TUA5*	*ACT11*	*RPL10A*	*ACT1*	*TUA4*	*Rubisco*	*MDH*	*ACT7*	*GAPDH*	*TUA2*	*EF-1-α*
RefFinder	*SAMDC*	*RPS25*	*F-BOX*	*PGK*	*RPL10A*	*TUA5*	*ACT1*	*TUA4*	*ACT11*	*Rubisco*	*MDH*	*ACT7*	*GAPDH*	*TUA2*	*EF-1-α*

Supplementary Table S3 Five sample sets from *Neolamarckia cadamba* used in this study.

Experimental sample sets	Tissue type	Number of treatments	Biological replicates	Sampling dates	Total number of samples (treatments * replicates * dates)
Different tissues	Roots, young leaves, Mature leaves, xylem, phloem	1	3	1	15
Different developmental stages	Mature leaves	1	3	4	12
Different photosynthetic cycle	Mature leaves	3	3	1	9
Drought treatment	Mature leaves	5	3	1	15
Cold treatment	Mature leaves	5	3	1	15

Supplementary Table S4 Characteristics of candidate reference genes and qRT-PCR primers in *Neolamarckia cadamba.* F represents forward primer while R represents reverse primer.

Gene symbol	GeneBank accession number (CDS)	GeneBank accession number (gDNA)	Annotation	Primer sequence (5’-3’)	Amplicon length (bp)
ACT1	KY922888	KY922871	Actin 1	F:GTCAAGGCTGGGTTTGCTGGGG<br>R:CCCAACATAAGCATCTTTTTGC	117
ACT7	KY922889	KY922872	Actin 7	F:AACTGGTATTGTCCTGGATTCTGG<br>R:CGGTGAGGATCTTCATGAGATG	104
ACT11	KY922890	KY922873	Actin 11	F:TGCTAGTGGTCGTACAACGG<br>R:ATCTGTAAGATCGCGCCCAG	133
TUA2	KY922891	KY922874	Tubulin alpha 2	F:ATACCATTGGCAAAGAGATCGT<br>R:GGACCCAAGACCAGATCCAGTG	131
TUA4	KY922892	KY922875	Tubulin alpha 4	F:CCCGTGGCCACTACAC<br>R:CAACAGGGATCCCAACC	149
TUA5	KY922893	KY922876	Tubulin alpha 5	F:CGGATGTTGTAGTGCTTTTG<br>R:TGGTTTGGAACTCGGTGA '	180
EF-1-α	KY922894	KY922877	Elongation factor 1-alpha	F:GCAACAAGATGGATGCCACCAC<br>R:GAACCCAGAGATGGGAACAAAG	134
Rubisco	KY922902	KY922879	Ribulose bisphosphate carboxylase	F: GATTAGTTTTGCGGGTTCT<br>R:TTCCACCGAGACGACCAG	143
RPS25	KY922900	KY922883	Ribosomal protein S25	F:CCCTCTGTTCTTTCCGACCG<br>R:GGCTTGCATGAGATGAGACC	109
RPL10A	KY922901	KY922884	Ribosomal protein L10A	F:TGGCGTTGCCTGTCTATCAG<br>R:CCGAACCTCGTGAATCCTCC	176
MDH	KY922902	KY922885	Malate dehydrogenase	F: TATTCTTCACGGTTTTCCTAC<br>R: GCTTCTTCTTGGTATCCTCA	157
GAPDH	KY922898	KY922881	Glyceraldehyde-3-phosphate dehydrogenase	F:TTGTCTCAAACCGCAACCCT<br>R:TTAGCTCCGGCTTGGATGTG	124
PGK	KY922899	KY922882	Phosphoglycerate kinase	F:GGTGTCACCCCGAAGTACAG<br>R:ATTCTCCAGGAGCAGCACAC	153
SAMDC	KY922897	KY922880	S-adenosylmethionine decarboxylase	F:GGGACCAACTGGATGAAA<br>R:TGTAAGGGAAGACAAAGAG	117
F-BOX	KY922895	KY922878	F-Box protein	F:CTTCCACTCCTCCCATCA<br>R:CAGACTTCATCAGTAGCAAAAC	149
